# Upgrading oxygenated Fischer-Tropsch derivatives and one-step direct synthesis of ethyl acetate from ethanol - examples of the desirability of research on simple chemical compounds transformations

**DOI:** 10.1186/s13065-014-0077-9

**Published:** 2014-12-19

**Authors:** Roman Klimkiewicz

**Affiliations:** Institute of Low Temperature and Structure Research, Polish Academy of Sciences, Okólna 2, Wrocław 50-422 Poland

**Keywords:** Fischer-Tropsch, Oxygenates, Ethyl acetate, Bioethanol, Primary alcohols, Biodiesel

## Abstract

Oxygenates formed as by-products of Fischer-Tropsch syntheses can be transformed into other Fischer-Tropsch derived oxygenates instead of treating them as unwanted chemicals. One-step direct synthesis of ethyl acetate from ethanol is feasible with the use of some heterogeneous catalysts. Despite their apparent simplicity, both transformations are discussed as targeted fields of research. Furthermore, the two concepts are justified due to the environmental protection. Arguments regarding the Fischer-Tropsch process are focused on the opportunities of the utilization of undesirable by-products. The effective striving for their utilization can make the oxygenates the targeted products of this process. Arguments regarding the one-step direct synthesis of ethyl acetate underline the environmental protection and sustainability as a less waste-generating method but, above all, highlight the possibility of reducing the glycerol overproduction problem. The production of ethyl acetate from bioethanol and then transesterification of fats and oils with the use of ethyl acetate allows managing all the renewable raw materials. Thus, the process enables the biosynthesis of biodiesel without glycerine by-product and potentially would result in the increase in the demand for ethyl acetate.

**Electronic supplementary material:**

The online version of this article (doi:10.1186/s13065-014-0077-9) contains supplementary material, which is available to authorized users.

**Graphical Abstract:**
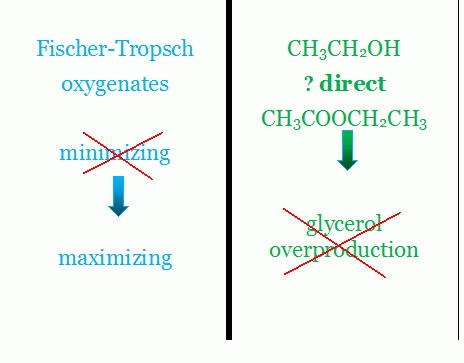
.

## Introduction

Oxygenates are organic oxygen compounds – common oxygenated hydrocarbon derivatives. Their definition is vague although intuitively obvious – e.g. simple ethers rather than sugars. Therefore, it can be thought that simple research on transformation of oxygenates is inexpedient as it has already been performed and is generally known while the research work has moved towards more advanced structures. It is true only to certain extent. The purposefulness of the studies on transformations of certain oxygenates into other ones is justified by dynamic changes in their interdependence. In order to visualize the purposefulness of studies on the catalytic conversion of oxygenates some examples are presented hereinafter.

Here is a simple model showing why even advanced studies concerning a simple issue require some modifications over time.

In the following sequence:

1 hydrocarbon→alcohol→aldehyde→esteroranyotherderivative

aldehydes are more highly-processed and more highly-oxidized products than alcohols. Numerous methods of oxidation or dehydrogenation of alcohols to aldehydes have been developed, especially the key ones, including catalysts, some of which are almost perfect. However, the answer to the question whether it is worth to obtain all aldehydes from alcohols or, in other words, whether it is worth to convert all alcohols into aldehydes, is “no”.

Accordingly, the research on the development of the catalytic transformations of simple oxygenates may have new motivation. The efforts to minimize the side formation of oxygenates as “unfavourable” for Fischer-Tropsch products may be abandoned in favor of the intentional exploitation of these compounds. Additionally, even uncomplicated methods of ethyl ethanoate synthesis can be replaced with even simpler, direct ones.

## Review

### Non-schematic catalytic transformations

Obtaining formaldehyde from methanol is an exemplary and important process in which the following sequence (1) trend is maintained. This process can be carried on silver or oxide catalysts. It can take place by dehydrogenation, oxidative dehydrogenation [1]-[3] or, optionally, by steam reforming [4]. However, seven decades ago, “oxo synthesis” or “oxo process“ occurred. It is a homogeneously catalysed hydroformylation process [5], i.e. the synthesis of aldehydes from alkenes and carbon monoxide. In particular, the hydroformylation of propylene can result in two isomeric products, butyraldehyde or isobutyraldehyde. This is the basic industrial synthesis of aldehydes. Thus, these aldehydes are cheaper than the alcohols related to them. As a consequence, these aldehydes are hydrogenated to alcohols, *n*-butanol and isobutanol [6]. Regular butyraldehyde, unlike isobutyraldehyde, is also transformed into 2-ethylhexanol in a sequence involving aldol condensation reaction followed by hydrogenation of the aldol product [6]. Isobutyraldehyde is sometimes regarded as a less valuable isomer [7]. A more versatile option is the tandem of hydroformylation and hydrogenation of alkene to alcohol [8].

The direction of conversion is opposite to that in sequence (1) - alcohols are obtained from aldehydes. Of course, there are many examples of new developments of reactions differing from the scheme of gradual oxidation: hydrocarbon into alcohol, alcohol into aldehyde, and so on:

- the above hydroformylation [9];

- Reppe reaction related to carbonylation is also used on the industrial scale [10];

- the Kucherov reaction, replaced by the Wacker process (also known as the Hoechst-Wacker process), i.e. method for the production of acetaldehyde via the direct oxidation of ethylene in an aqueous medium [11];

- acetic acid is obtained on the industrial scale through the carbonylation of methanol obtained from the synthesis gas (Monsanto process) [12];

- this family includes also a group of hydrocarbonylation reactions, i.e. homologation [13]. The homologation reaction is any chemical reaction that converts the reactant into the next member of the homologous series that differs by a constant unit, generally the methylene group. An example of this is the synthesis of higher alcohols [14],[15];

- in turn, the family of bimolecular condensations includes, inter alia, the Guerbet reaction, which consists in converting a primary aliphatic alcohol into its β-alkylated dimer alcohol accompanied by dehydration, e.g. ethanol to *n*-butanol, n-butanol to 2-ethylhexanol [16]-[24].

The purpose of the above examples is to illustrate the diversity of the seemingly similar situations and their specificity as well as to prove that "the only correct" direction of transformation of oxygenates does not exist.

Hence, the purposefulness of the work on any particular transformation of oxygenates depends on a whole range of market factors. First of all, this work comes down to the development of an active and selective catalyst. The complexity of such an undertaking includes important concepts concerning the catalysis. Many factors are significant at the same time. There is no point in discussing them individually here or even in trying to rank them. Such complexity indicates that designing the optimal solution for a specific, desired transformation, i.e. both preparation of a catalyst and the course of this transformation, will not facilitate the execution of other tasks, even closely related ones, because of the multitude of significant factors and the multitude of interdependencies. The variation of such interdependencies does not have to be linear so extrapolations and interpolations are not always effective. Another conclusion is that the number of factors to define a specific process excludes a random selection of catalyst components.

It may happen that the understanding of phenomena brings useful generalizations. In the 1920s Taylor deduced that since a catalyst can be completely poisoned by such a little amount of poison that it is able to cover only a small fraction of the catalyst surface it means that the active participants in the reaction are only certain groups of atoms on the surface [25]. In such a concept the active centres must differ from each other to meet different requirements. The same groups of atoms do not have to be active centres of various transformations. In another reaction, considering different requirements of reagents, other groups of atoms can perform the functions of centres on the same surface. There are many types of centres but their definitions are not entirely consistent. Apart from proving their presence and identifying their type it is advisable to estimate their number and power. The catalytic act itself may take different courses [26]. The basic generalizations include the Langmuir-Hinshelwood model from the 1920s where both reagents activated with adsorption on the surface reacted with each other, the Eley-Rideal model from the 1930s where one of the reagents adsorbed on the surface became an easy target for another reagent attacking from the atmosphere, and the Mars-van Krevelen model from the 1950s in which the catalyst’s oxygen participated actively and the gap after it had to be filled in to enable the next act.

The above differentiated mechanisms relate to the catalyst per se. Catalytically active materials are often affixed to the catalyst support due to their high surface area and saving of the valuable materials. Additionally, quite a while ago, the boundary between the actual catalyst and the catalytic support began to blur. Tauster found the strong metal-support interaction (SMSI effect) [27] predicted much earlier by Schwab [28].

### Fischer-Tropsch derived oxygenates

Coming back to the main problem of the dependence of the justification of transformation of oxygenates on variable conditions, a new attitude regarding the products of the Fischer-Tropsch synthesis should be noted. The F-T synthesis is a technology developed in the 1920s, which consists in producing liquid fuels from any carbon materials other than petroleum. This is a technology related to that used much earlier than F-T, in tar and charcoal manufactories by tar makers and charcoal burners. Disregarding the details of gas names definitions (wood distillation gas, generator gas, coke-oven gas, town gas, water gas, or synthesis gas) from any carbon material: wood, coal, biomass, natural gas [29]-[32] (including shale gas [33]), and even better with the participation of water vapour, sometimes also oxygen, it is possible to obtain gases containing carbon monoxide and hydrogen in various proportions and also other components as a result of gasification, reforming or pyrolysis. It is possible to separate hydrogen from such gases and they are suitable for syntheses of hydrocarbons, i.e. liquid fuels but also for chemical syntheses, e.g. syntheses of methanol and higher alcohols [34]-[41]. Alcohols can be the source of hydrogen after dehydrogenation. The alcohols may also be subject to transformations to other oxygenates or hydrocarbons.

During such syntheses, oxygenates are formed as by-products quantitatively and qualitatively depending on numerous factors [42]-[54]. These relationships have usually been the subject of research. Especially important is the impact of the presence of oxygenates on the course of the process and possibility to manipulate the product spectrum by selective addition of oxygenates [55]-[61]. Therefore, there are two possibilities: treat them as unwanted products [62]-[64] and try to reduce their amount or to strive for their utilization [65]-[73], also by the use of new technologies, such as membrane separations [74]-[76]. Oxygenates can be transformed into other oxygenates or into hydrocarbons through hydrogenation. Two important coupling reactions for the upgrading of monofunctional oxygenated compounds are ketonization and aldol condensation/hydrogenation [77],[78]. The conversion of oxygenates into hydrocarbons, which are lower-processed products, may seem pointless. However, it may be an attractive direction if we have redundant oxygenates [42],[79] and if we avoid other expensive treatments such as separation or costly attempts to improve selectivity in this way. There is also the option of steam reforming [80]. Thus, oxygenates need not be a nuisance. They can be seen as a source of intermediates for value-added chemicals [81]-[85].

It is a popular opinion that the Fischer-Tropsch technology has survived only in the RSA because of the isolation of this country. This is truth but not ultimate. This technology has been developed, inter alia, in the United States, Uzbekistan, Russia and China as an alternative technology [33]. Thus, the current uproar about the underground coal gasification is much late. Since 1944 a few large plants for the F-T process have been built in the United States under the Synthetic Liquid Fuels program and they still exist as a form of strategic reserve [86].

There are several reasons for the renaissance of the Fischer-Tropsch technology. One of them is the return to the abandoned idea of using hydrogen for transport but in a different aspect. Steam reforming of natural gas, i.e. the equivalent of the coal gasification process as well as the coal gasification itself can be the sources of hydrogen. Also other chemical processes such as catalytic processes of dehydrogenation of alcohols and WGSR (Water Gas Shift Reaction) can be the sources of hydrogen.

The WGSR is the most important side reaction of the Fischer-Tropsch synthesis [87],[88] of hydrocarbons or methanol from – it should be reminded here – carbon monoxide and, after all, hydrogen. The whole point is that water is also the product of these transformations and WGSR allows hydrogen recovery. Of course, WGSR is also used as an independent reaction, unrelated to F-T.

– The increase in demand for methanol is also an important trigger. In the chemical industry worldwide there is no other product with such a mass and multi-directional use as methanol. It is an irreplaceable intermediate product for the syntheses of many chemicals, in particular formaldehyde and various related products. Methanol is also the main base for the production of top quality petrol and diesel fuels. It can be added, blended, in significant volumes, directly to petrol fuels [89] or transformed into dimethyl ether [90], a diesel fuel substitute crucial for the Methanol-to-Gasoline (MTG) and Methanol-to-Olefin (MTO) processes. The use of methanol as a component in the transesterification of triglycerides to yield a form of biodiesel [91] may become widespread to the extent similar to obtaining ethanol from biomass. Methanol is already used today on a large scale (the global demand for methanol is around 60 million ton per year [92]) and the forecasts for the average annual growth in the methanol production are estimated to be high. Along with the development of fuel cells, methanol will become a strategic resource. The Blasiak’s concept is therefore constantly evolving [93]-[97].

– The reason is also the return to the concept of using heavier fuels of natural origin. It is also a comeback, because the first diesel engine built by Rudolf Diesel was fuelled by peanut oil and such was the original intention.

Despite these facts, the preparations for the return of the Fischer-Tropsch technology proceeded slowly. This was explained by the adverse financial situation resulting from the significant decline in oil prices, which reduced the competitiveness of coal fuels in relation to petroleum fuels. The current price fluctuations emphasize the purposefulness of long-term studies in this field. May the dim prospects for shale gas not overshadow this opportunity and not be the cause of another failure to develop chemical technologies based on coal resources.

### Ethyl acetate from ethanol

Another example of a new approach to transformation of a simple oxygenate is the concept of a single-stage production of ethyl acetate. Of course, the idea of primary alcohols transformation into esters had to appear during the development of organic chemistry [98] as a result of the next step of dehydrogenation of alcohols and of the earlier step of Tishchenko reaction. However, publications on the one-step direct synthesis of esters from primary alcohols with the use of heterogeneous catalysts are not numerous and are of technical reports character. Nevertheless, among the patented methods of ethyl acetate production only the achievement of the Davy Process (Kvaerner Process Technology, Sasol Chemical Industries) [99] is close to the idea. For instance, the process of Chinese National Petroleum, described in the Chemical Weekly [100], is related to a one-step process of transformation of ethanol into ethyl acetate; however, by the partial oxidation of ethanol to acetic acid and successive esterification by the excess of ethanol. In relation to other more complicated transformations, this relatively simple one is described rather poorly [76],[101]-[118]. Of course, there is a different option that illustrates the variability of the research directions – as mentioned at the beginning – the elaboration of ethyl acetate hydrogenation to ethanol [119],[120].

While observing that literature announcements on primary alcohols conversion over metal oxide containing heterogeneous catalysts are focused on the dehydration to ethers and alkenes or dehydrogenation to aldehydes, the both reactions are so popular that may be even treated as tests for acidity and basicity of the surface. However, some catalysts are able to perform a secondary condensation of created aldehydes to ketones containing 2*n*-1 carbon atoms in aliphatic chain [55],[56],[121]-[125], where *n* stands for the number of C atoms in *n*-alcohol undergoing the conversion. These catalysts are capable of triggering the following reaction chain:

2 $$2\;RCH_{2}OH\;\to \;2\;\mathrm{RCHO}\;\to \;\mathrm{RCOOC}H_{2}R\;\to \;\mathrm{RCOR}$$

In the reports in literature about this group of transformations there is a large divergence of views as to the route of the reaction. Among others, there are suggested pathways via aldol, via aldol and secondary alcohol, simultaneously via ester and aldol, via hemiacetal and ester, or aldol with a parallel hydrogen transfer reaction [126]-[128]. This secondary reaction is not always intentional [129]. During the alkylation of phenol [130] with alcohols higher than methanol the competitive reaction can occur: bimolecular condensation of primary alcohols to ketones containing 2n-1 carbon atoms [124],[131],[132]. Ketonization of primary alcohols, and consequently aldehydes and esters, allows the synthesis of symmetrical and unsymmetrical ketones [133],[134] and expands possibilities of utilization of such materials [134],[135]. This transformation is akin to the bimolecular ketonization of monocarboxylic acids [136]-[141], which is much more frequently described in the literature. The suitable oxide catalysts are applied in the bulk form or are often deposited on various supports, including materials of natural origin [142]-[144].

Most of the catalysts of dehydrogenative character transform primary alcohols only to aldehydes and less often to symmetric ketones. Excessive overrun of conversion optimal conditions leads to possible degradation products. Nevertheless, the proper selection of control parameters allows obtaining esters as major conversion products. Obviously, such a termination of the transformation at the ester stage is effective only with respect to specific catalysts.

The important incentive, impetus to intensify the research on the termination of the course of bimolecular condensation of primary alcohols in the ester phase is the potential increase in the ethyl acetate demand. The major driving force for that demand originates from the search for new energy sources which would be renewable and “clean” – not causing additional carbon dioxide emission. Biomass derived fuels meet these expectations and can partly replace fossil fuels. Diesel oil substitute – biodiesel – is one of them. Ethyl acetate can be used in some methods of biodiesel production. Biodiesel synthesis with the use of ethyl acetate is an attractive option because of the possibility of reducing the glycerol overproduction problem [145]. The majority of methods of the conversion of fatty materials (including rapeseed oil) to fuel components is fraught with side formation of glycerol, according to the scheme:

3 triglyceride+alcohol→fattyacidester+glycerol

Glycerol overproduction as well as the problems with its utilization initiate the search for new methods of its management. The use of ethyl acetate instead of alcohol in the process of cross transesterification of triglycerides in the Gliperol process is an example of such projects [146].

In this case the transformation of fats follows the scheme:

4 triglyceride+ethylacetate→fattyacidethylester+glyceroltriacetate

Glycerol triacetate (triacetin) does not require the separation from the final product as it improves biofuel combustion conditions [147]. Besides, ethyl esters have better fuel properties than methyl esters (including higher heating value, incomplete combustion products free from formaldehyde, a lower freezing point) [148]. The transesterification of fats and oils with the use of ethyl acetate allows managing all the renewable raw materials (including glycerine) for fuel production. It is therefore a very attractive method for the manufacture of the fuel components. However, one of the factors restricting its wider application is ethyl acetate deficiency in the market (ethyl acetate is widely used in industry [149] and the production methods have some disadvantages [150]-[152]: multistage processes and necessity of the catalyst separation require a complicated system of tanks and equipment, raw materials cause corrosion, whereas acetaldehyde is strongly toxic (Fischer esterification – catalyst: sulfuric acid, *p*-toluenesulfonic acid, Tishchenko condensation of acetaldehyde – catalyst: aluminum triethoxide, condensation of acetic acid with ethylene – catalyst: bentonites, heteropolyacids)).

Changes in the fuel market, including the strong drive towards the idea of using ethanol and, consequently, a large increase in the production of bioethanol, are consistent with this concept. Thus, ethyl alcohol can be a cheap, renewable resource for the production of ethyl acetate [153]. The concept of using ethyl acetate in the process of transesterification of oils and fats is also consistent with the increased production of rapeseed oil and with the increase in demand for transesterified oils. In addition, besides the potential demand for ethyl acetate and the availability of bioethanol, the technical simplicity of implementation of such a transformation due to few media and accompanying materials and the reduction of the amount of noxious by-products as compared to traditional methods of ethyl acetate production should be emphasized. Such a solution is convergent with ecological trends towards new technologies in order to increase the share of renewable materials in the overall fuel balance. Moreover, the method meets the requirements of green chemistry and is consistent with the principles of sustainable development.

## Conclusions

Research on the development of catalytic transformations of simple oxygenates is still justified. New market conditions make the production and processing of oxygenates as part of the Fischer-Tropsch synthesis purposeful. On the other hand, the increase in the potential demand for ethyl acetate against the background of high availability of ethanol justifies further research on a direct one-step catalytic synthesis of this ester.

## Authors’ original submitted files for images

Below are the links to the authors’ original submitted files for images. Authors’ original file for figure 1
