# Supplementary figures and images for: Upgrading oxygenated Fischer-Tropsch derivatives and one-step direct synthesis of ethyl acetate from ethanol - examples of the desirability of research on simple chemical compounds transformations
Source: Chem Cent J. 2014 Dec 19;8:77. doi: 10.1186/s13065-014-0077-9 (PMC4303706; doi:10.1186/s13065-014-0077-9)

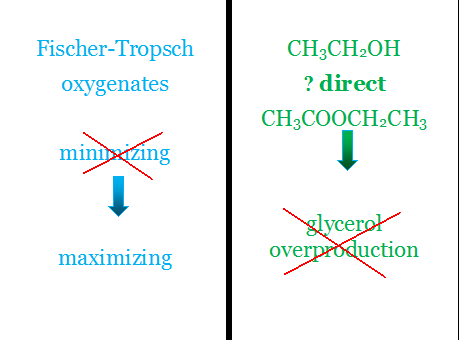

Supplement: Supplementary file 1 — Authors’ original file for figure 1 [file 13065_2014_77_MOESM1_ESM.png]
